# Surveillance of the prevalence, antibiotic susceptibility, and genotypic characterization of invasive candidiasis in a teaching hospital in China between 2006 to 2011

**DOI:** 10.1186/1471-2334-13-353

**Published:** 2013-07-30

**Authors:** Fang Li, Lin Wu, Bin Cao, Yuyu Zhang, Xiaoli Li, Yingmei Liu

**Affiliations:** 1 Department of Infectious Diseases and Clinical Microbiology, Beijing Chao-Yang Hospital, Capital Medical University, Beijing 100020, China

**Keywords:** Invasive *Candida*, Epidemiology, Antifungal susceptibility, Genotyping

## Abstract

**Background:**

Invasive candidiasis is an important nosocomial infection associated with high mortality among immunosuppressive or critically ill patients. We described the incidence of invasive candidiasis in our hospital over 6 years and showed the antifungal susceptibility and genotypes of the isolated yeast.

**Method:**

The yeast species were isolated on CHROMagar *Candida* medium and identified using an yeast identification card, followed by analysis of the D1/D2 domain of 26S rDNA. The susceptibilities of the isolates to flucytosine, amphotericin B, fluconazole, itraconazole, and voriconazole were tested using the ATB FUNGUS 3 system, and that to caspofungin was tested using E-test strips. *C. albicans* was genotyped using single-strand conformation polymorphism of CAI (*Candida albicans* I) microsatellite DNA combined with GeneScan data.

**Results:**

From January 2006 to December 2011, a total of 259 isolates of invasive *Candida* spp. were obtained from 253 patients, among them 6 patients had multiple positive samples. Ninety-one stains were from blood and 168 from sterile fluids, accounting for 6.07% of all pathogens isolated in our hospital. Most of these strains were *C. albicans* (41.29% in blood/59.06% in sterile body fluids), followed by *C. tropicalis* (18.06%/25.72%), *C. parapsilosis* (17.42%/5.43%), *C. glabrata* (11.61%/3.99%) and other *Candida* spp. (11.61%/5.80%). Most *Candida* spp. were isolated from the ICU. The new species-specific CLSI *candida* MIC breakpoints were applied to these date. Resistance to fluconazole occurred in 6.6% of *C. albicans* isolates, 10.6% of *C. tropicalis* isolates and 15.0% of *C. glabrata* isolates. For the 136 *C. albicans* isolates, 54 CAI patterns were recognized. The *C. albicans* strains from blood or sterile body fluids showed no predominant CAI genotypes. *C. albicans* isolates from different samples from the same patient had the same genotype.

**Conclusions:**

Invasive candidiasis has been commonly encountered in our hospital in the past 6 years, with increasing frequency of non*-C. albicans*. Resistance to fluconazole was highly predictive of resistance to voriconazole. CAI SSCP genotyping showed that all *C. albicans* strains were polymorphic. Invasive candidiasis were commonly endogenous infection.

## Background

In the early 1980s, fungi have emerged as major causes of human disease, especially in immunocompromised and hospitalized patients [1]. *Candida* spp. are the fourth most common pathogens in nosocomial bloodstream infections (BSIs), and they account for 12% of all hospital-acquired BSIs in the United States [2]. The mortality of candidemia in adults and neonatal children was estimated to be 15%–25% and 10%–15% in the United States, respectively [3]. Further, studies have shown that *Candida* infection can extend the duration of hospital stay [4], increase the cost of medical care [5], and lead to a high mortality (30%–40%) [6]. In recent years, non-*C. albicans* spp. have been detected more commonly in candidemia [7,8]. Both *C. albicans* and non-*C. albicans* cause invasive candidiasis, and the antifungal resistance of invasive *Candida* aggravates the situation. Some reports on *Candida* resistance to antifungals showed that resistance could make therapy more difficult [9,10]. The species and antifungal resistance of *Candida* differ among geographies, and it is necessary to survey the prevalence and drug resistance of *Candida* spp. in different locations in order to ensure effective antifungal therapy against invasive *Candida*.

Few studies on the long-term monitoring of the incidence and antifungal susceptibilities of invasive *Candida* spp. have been reported from China. This study aims to describe the trend of invasive candidiasis in a teaching hospital during the last 6 years.

## Methods

### Study population and data collection

The study was conducted in Beijing Chaoyang Hospital, a teaching facility of the Capital Medical University in Beijing, China. According to the diagnostic criteria, all patients with invasive candidiasis were enrolled from different wards from 2006 to 2011. The following information was collected including age, sex, underlying diseases, et al. This investigation had the approval of the Ethics Committee of Beijing Chaoyang Hospital and informed consent was obtained from all study subjects or their next of kin.

### *Candida* strain collection

A total of 259 isolates of invasive *Candida* spp. were obtained from 253 patients, among them 6 patients had multiple positive samples. Invasive *Candida* strains were isolated from blood (91) and sterile samples (168) (including ascitic fluid, bile,central venous catheter, pleural fluid); the total of 259 samples accounted for 6.07% of all pathogens (5.75% of 1583 from blood and 6.27% of 2684 pathogens from sterile fluids) isolated in Beijing Chao-yang Hospital during the study period. Patients come from 13 provinces in china, mainly in northern china (including Beijing, Shanxi, Inner Mongolia, Heilongjiang et al.) Most of the *Candida* spp. were isolated from patients in the intensive care unit (ICUs). The *Candida* strains were primarily cultured on CHROMAgar *Candida* medium (JinZhang, Tianjing China) and identified using an yeast identification card (BioMérieux, France) or the API 20C AUX system (BioMérieux, France), followed by sequence analysis of the D1/D2 domain in the 26S rDNA [11]. Quality control isolates included *C. albicans* ATCC90028, *C. parapsilosis* ATCC22019, and *C. krusei* ATCC6258.

### Antifungal drug susceptibility test

A total of 240 *Candida* strains were tested for antifungal susceptibility. The susceptibility of these strains to 6 antifungal drugs was tested *in vitro*. Flucytosine, amphotericin B, fluconazole, voriconazole, and itraconazole were tested using the ATB FUNGUS 3 system (1000723260; BioMérieux, France), and caspofungin, using the E-test (BJ3625 AB Biodisk; Solna, Sweden). For fluconazole, voriconazole, and itraconazole, the minimal inhibitory concentration (MIC) was determined by determining the concentration at which a prominent reduction in the yeast cell count was observed after 24 h of treatment. For the caspofungin, the MIC was determined by detection of a significant reduction in growth after 24 h of incubation. The MIC for amphotericin B and flucytosine were defined as the lowest concentration at which no visible growth was detected. Recently approved CLSI 24 h resistance breakpoints for fluconazole,voriconazole and echinocandins were used [12]: isolates of *C. albicans*, *C. tropicalis* and *C. parapsilosis* with an MIC ≥ 8 μg/ml and isolates of *C. glabrata* with an MIC ≥ 64 μg/ml were considered resistant to fluconazole; isolates of *C. albicans, C. tropicalis* and *C. parapsilosis* with an MIC ≥ 1 μg/ml were considered resistant to voriconazole; isolates of *C. albicans* and *C. tropicalis* with an MIC ≥ 1 μg/ml were considered resistant to caspofungin, isolates of *C. parapsilosis* with an MIC ≥ 8 μg/ml were considered resistant to caspofungin, isolates of *C. glabrata* with an MIC ≥ 0.5 μg/ml were considered resistant to caspofungin. Data were reported as the MIC ranges; the MIC_50_ and MIC_90_ values; and the number of resistant (R) isolates.

### DNA extraction and PCR

DNA extraction and PCR amplification of CAI microsatellite DNA were performed according to the methods of [13]. The microsatellite locus CAI was amplified by PCR (forward primer, 5′-ATG CCA TTG AGT GGA ATT GG-3′; reverse, 5′-AGT GGC TTG TGT TGG GTT TT-3′). For GeneScan analysis, the forward primer was fluorescently labeled with 6-carboxyfluorescein at the 5′ end. PCR amplification was performed in a thermocycler (ICycler; Bio-Rad, Hercules, CA) with the following program: initial denaturing step at 95°C for 4 min; 33 cycles of denaturation at 95°C for 30 s, annealing at 60°C for 30 s, and extension at 72°C for 1 min; and a final extension step at 72°C for 7 min.

### Genotype analysis

Genotype analysis of the amplified CAI fragments was carried out using the methods described by [14]. The sizes of the PCR products were automatically determined using the ABI 370 genetic analyzer (Applied Biosystem, Foster City, CA). Because of the diploid nature of *C. albicans*, the CAI genotypes of the strains were designated by the number of trinucleotide repeat units in both alleles of the microsatellite locus [14].

### Statistical analysis

Drug susceptibility testing was analyzed using WHONET 5.6. Data were analyzed with SPSS 17.0 (SPSS Inc., Chicago, IL). Categorical variables were compared using the chi-square or Fisher’s exact test. A P value of 0.05 was considered significant.

## Results

### Patient characteristics

According to the diagnostic criteria of invasive candidiasis [15], a total of 253 patients were enrolled: male (170/253) 67.19%, female (83/253) 32.81%. The mean age was 65.08 ± 13.87 ys. The incidence rate of invasive candidiasis was 0.25 cases per 1,000 admissions from 2006–2011. The mortality rate at 6 weeks in ICUs (39.73%) was higher than that in other wards (17.39%), P < 0.05. The types of infection were bloodstream infection (approximately 35%), abdominal infection (approximately 35%), biliary tract infection (approximately 18%), catheter related blood stream infection (approximately 7%), and chest infection (approximately 6%), respectively.

### Distribution of *Candida*

**Figure 1:**
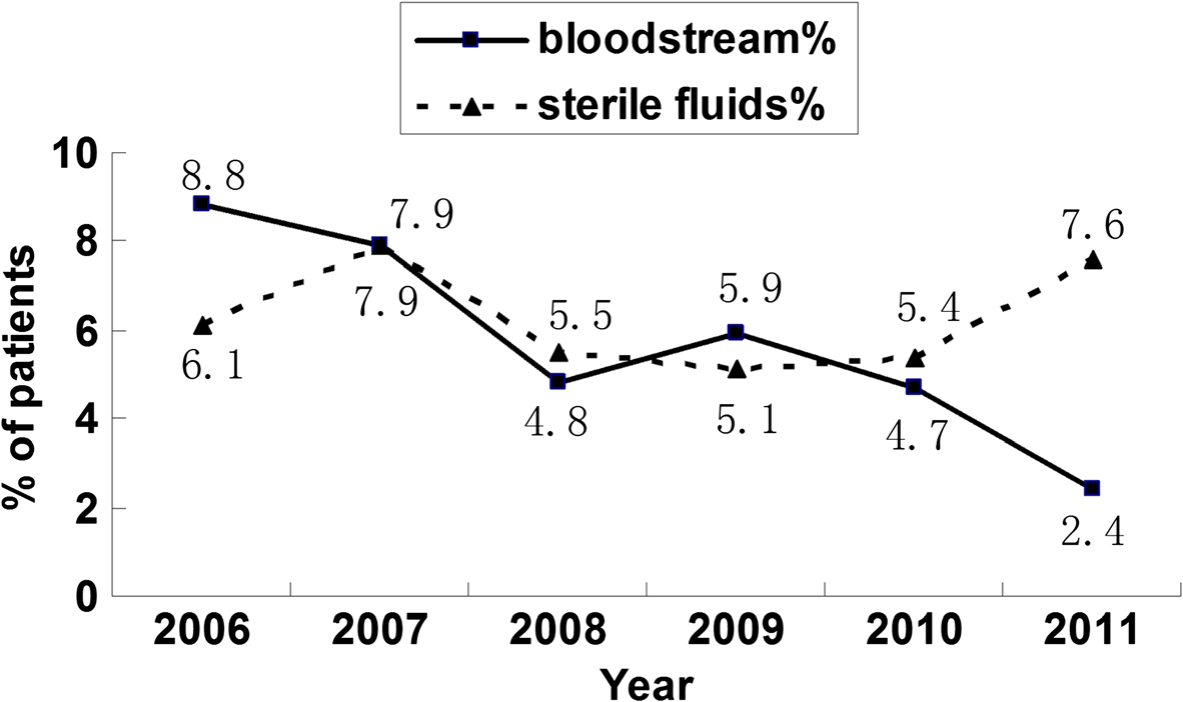
**Changes in the isolation rate of *Candida *spp. in the last 6 years.** The annual rates of invasive candidiasis from 2006 to 2011 were showed in the figure, according to the identification of *Candida* strains from bloodstream and sterile fluids (ascitic fluid, bile, central venous catheter and pleural fluid).

**Figure 2:**
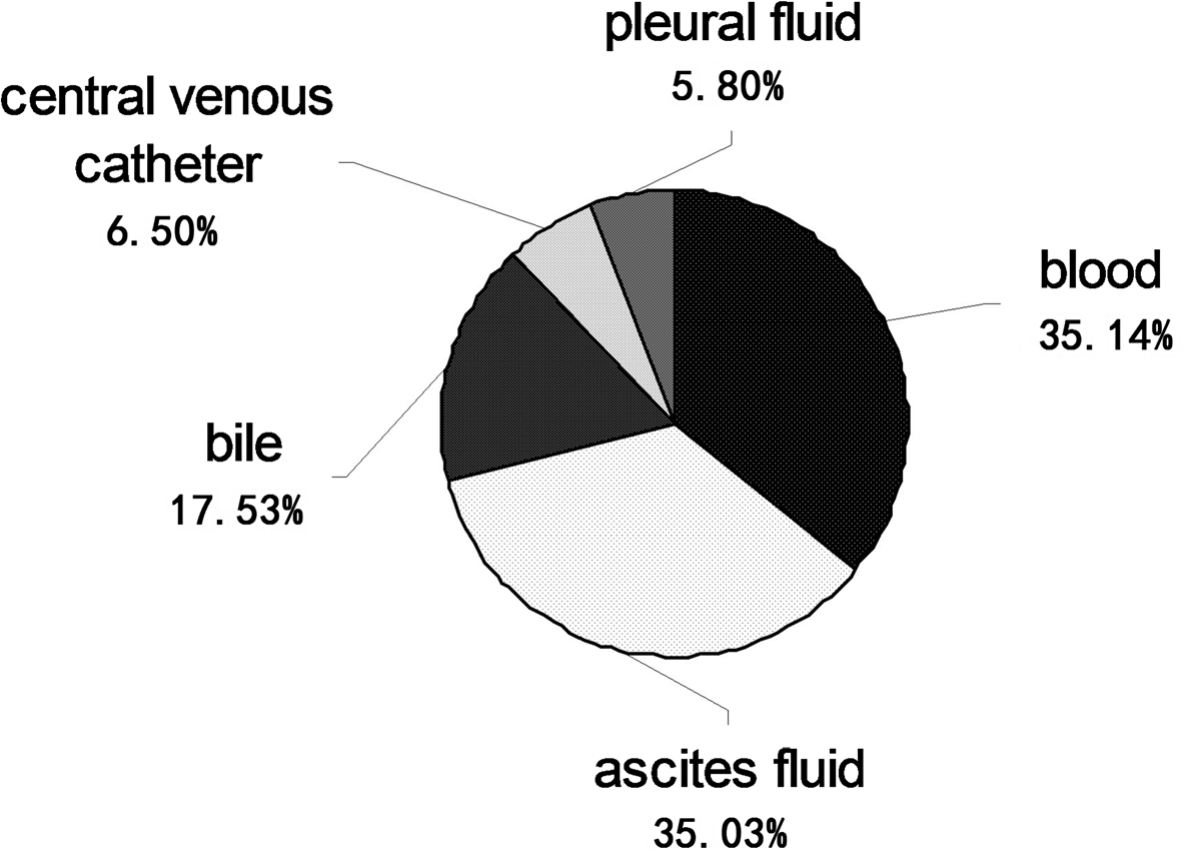
**Distribution of *Candida *spp. in different samples (n = 259).** The distribution of the 259 invasive candidiasis specimens was as followed: blood 35.14%, ascitic fluid 35.03%, bile 17.53%, central venous catheter fluid 6.50%, and pleural fluids 5.80%.

**Figure 3:**
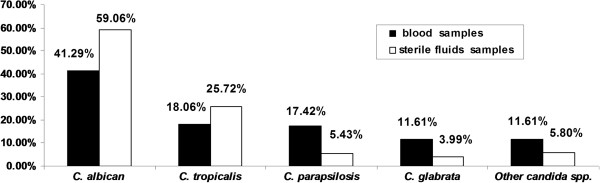
**Distribution of *Candida *spp. in blood and sterile fluids samples.** Most of these strains were *C. albicans* (41.29% in blood/59.06% in sterile fluids), followed by *C. tropicalis* (18.06%/25.72%), *C. parapsilosis* (17.42%/5.43%), *C. glabrata* (11.61%/3.99%) and other *Candida* spp. (11.61%/5.80%).

A total of 91 *Candida* strains were isolated from blood, which account for 5.75% of the 1583 pathogens isolated from blood from January 2006 to December 2011. The annual rates of candidemia over the 6 years of the study were as follows: 8.8%, 7.9%, 4.8%, 5.9%, 4.7%, and 2.4%. Further, 168 *Candida* strains were collected from sterile fluids (ascitic fluid, bile, central venous catheter and pleural fluid), which accounted for 6.27% of the 2684 pathogens isolated from sterile fluids (Figure 1). The distribution of these 259 invasive candidiasis specimens was as follows: blood 35.14%, ascitic fluid 35.03%, bile 17.53%, central venous catheter fluid 6.50%, and pleural fluids 5.80% (Figure 2). Most of these strains were *C. albicans* (41.29% in blood/59.06% in sterile body fluids), followed by *C. tropicalis* (18.06%/25.72%), *C. parapsilosis* (17.42%/5.43%), *C. glabrata* (11.61%/3.99%) and other *Candida* spp. (11.61%/5.80%) (Figure 3).

The top 3 wards in which candidemia occurred were the ICUs (43.23%) and the hematology (16.13%) and the urology (10.32%). The wards in which *Candida* spp. were most frequently isolated from sterile fluids were the ICUs (41.91%) and the interventional radiology (18.15%) and the hepatobiliary (10.56%). Thus, our data showed that most patients with nosocomial candidasis were from the ICUs.

### Susceptibility of antifungal drugs

The susceptibilities of the 240 yeast strains to the 6 antifungal drugs are shown in Table 1. Most *Candida* strains were sensitive to all the antifungal drugs, although *C. glabrata* and *C. tropicalis* showed decreased sensitivity to triazole drugs (less than 90%). The percentages of isolates in the R categories were 7.5% for fluconazole and 5.4% for voriconazole, respectively. Caspofungin and amphotericin B exhibited excellent antifungal activity against all *Candida* species. The MIC of fluconazole was ≥8 μg/ml for *C. albicans* isolates (6.6%) and that of voriconazole was ≥1 μg/ml (2.9%). Further, the MIC of fluconazole was ≥8 μg/ml for *C. tropicalis* isolates (10.6%) and that of voriconazole was ≥1 μg/ml (10.6%). The MIC of fluconazole was ≥64 μg/ml for *C. glabrata* isolates (15.0%). Three of 136 *C. albicans* isolates (approximately 2%) were resistant to both FLC (MIC ≥ 8 μg/ml) and VRC (MIC ≥ 1 μg/ml). Six of 59 *C. tropicalis* isolates (approximately 10%) had FLC MIC ≥ 8 μg/ml and VRC ≥ 1 μg/ml. Three of 20 *C. glabrata* isolates (approximately 15%) had FLC MIC ≥ 64 μg/ml and VRC ≥ 8 μg/ml. No isolate was resistant to all existing classes. Cross-resistance to triazole antifungal drugs was found in 2.9% *C. albicans* and 10.6% *C. tropicalis* isolates. Resistance to fluconazole was highly predictive of voriconazole resistance. No statistically significant differences in azole resistance (P > 0.05) were observed for *C. albicans*, *C. tropicalis*, and *C. glabrata* isolated from either blood or sterile fluids.

**Table 1:** ***In vitro *antifungal susceptibility testing of 240 clinical isolates to 6 antifungal agents**

**Species (No of isolates)**	**Antifungal agent**	**MIC (μg/ml)**			**% Resistant**
		**Range**	**MIC**_**50**_^**a**^	**MIC**_**90**_^**a**^	
All isolates (240)	Fluconazole	1– > 128	1	4	7.5
	Voriconazole(except *C. glabrata)*	0.06– > 8	0.064	0.25	5.4
	Itraconazole(limit *C.albicans* only*)*	0.125– > 4	0.125	0.5	4.9
	Caspofungin^b^	0.032–2	0.125	0.5	0
	Flucytosine	< 4–16	4	4	
	Amphotericin B	< 0.5–0.5	0.5	0.5	
*C. albicans* (136)	Fluconazole	1– > 128	1	2	6.6
	Voriconazole	0.06– > 8	0.064	0.064	2.9
	Itraconazole	0.125– > 4	0.125	0.125	4.9
	Caspofungin^b^	0.032–0.38	0.094	0.19	0
	Flucytosine	< 4–16	4	4	
	Amphotericin B	< 0.5–0.5	0.5	0.5	
*C.tropicalis* (59)	Fluconazole	1– > 128	1	128	10.6
	Voriconazole	0.06– > 8	0.064	8	10.6
	Itraconazole	0.125– > 4	0.125	4	
	Caspofungin^b^	0.032–0.38	0.094	0.19	0
	Flucytosine	< 4–4	4	4	
	Amphotericin B	< 0.5–0.5	0.5	0.5	
*C. paraps-ilosis* (25)	Fluconazole	< 1–2	1	1	0
	Voriconazole	< 0.06–0.25	0.064	0.064	0
	Itraconazole	< 0.125–0.25	0.125	0.125	
	Caspofungin^b^	0.38–2	1	1.5	0
	Flucytosine	< 4– > 16	4	16	
	Amphotericin B	< 0.5–0.5	0.5	0.5	
*C.glabrata* (20)	Fluconazole	1–64	2	64	15.0
	Voriconazole	0.06–8	0.125	8	
	Itraconazole	0.125– > 4	0.5	4	
	Caspofungin^b^	0.047–0.38	0.19	0.25	0
	Flucytosine	< 4–4	4	4	
	Amphotericin B	< 0.5–0.5	0.5	0.5	

Footnote: ^a^MIC_50_, 50% minimal inhibitory concentration; MIC_90_: 90% minimal inhibitory concentration. ^b^Caspofungin tested using E-test strips.

### CAI genotypes of *C. albicans*

Genotype analysis was conducted for all 136 *C. albicans*, 54 CAI patterns were recognized. The overall frequencies of the dominant genotypes were as follows: 18–26, 7.40%; 16–21, 6.48%; 21–21, 5.55%; 11–11, 5.55%; 26–26, 4.63%; 25–33, 4.63%; 11–20, 4.46%; 11–18, 3.70%; and 18–18, 3.70%. In 9 cases, the genotypes of *C. albicans* isolates from different samples (blood, catheter fluid, ascitic fluid, and bile) from the same patient were identical.

## Discussion

This study described the distribution, *in vitro* antifungal susceptibilities, and genotyping results of invasive *Candida* spp. isolated between 2006 and 2011 from Beijing Chaoyang hospital in China.

The results showed that invasive candidiasis has frequently occurred in our hospital in the past 6 years. The average isolation rate of *Candida* spp. was 6.07% among all pathogens isolated from blood and sterile fluid samples. In most cases, the invasive candidiasis occurred in the ICUs, indicating that more attention must be paid to ICU patients in order to prevent them from contracting this disease. Although *C. albicans* was the most predominant species, the frequency at which non-*albicans Candida* were detected increased obviously over the study period. In our hospital, in more than half the recent cases of candidemia, the causative organisms were non-*albicans Candida* strains, *C. tropicalis, C. parapsilosis*, and *C. glabrata* were the most common among the non-*albicans Candida* spp. detected, in accordance with reports by Pereira GH, et al. [16,17]. In the case of the blood samples, *C. albicans* no longer comprised the majority of isolated but remained the most frequently isolated species (41.29%), followed by *C. tropicalis* (18.06%), *C. parapsilosis* (17.42%), and *C. glabrata* (11.61%). In the case of the sterile samples, *C. albicans* was the most commonly isolated organism (59.06%), followed by *C. tropicalis* (25.72%), *C. parapsilosis* (5.43%), and *C. glabrata* (3.99%). It is reported that *C. glabrata* is No.4 among the most isolated species, which are similar to our findings, but different from those reported from Europe and the US [1,18,19].

*C. abicans* has traditionally been the leading cause of candidemia worldwide, with the proportion of *C.abicans* infections decreasing and proportion of non-*C.abicans* infections increasing, especially *C. tropicalis*, *C. parapsilosis and C. glabrata.* The trend has also been observed in this study, the proportion of *C. abicans* candidemia cases decreased from 54% to 41%. At the same time, the proportion of *C. parapsilosis* candidemia, *C. glabrata* candidemia and *C. tropicalis* candidemia cases rose from 12% to 17%, 7% to 12% and 15% to 18%, respectively. In the ARTEMIS DISK global antifungal surveillance project undertaken between 1997 and 2007, 90% of infections were found to be caused by 4 *Candida* species, i.e., *C. albicans*, *C. glabrata*, *C. parapsilosis*, and *C. tropicalis*[20]. This distribution is similar to our study, *C. abicans, C.tropicalis,C. parapsilosis and C. glabrata* still comprise 92.67% of all isolates, that has not substantially changed in 20 years in the US [21]. During the last decade, the frequency of detection of the latter 3 species has been increasing in many countries, including China [22-24]. The widespread use of fluconazole may change the epidemiology of infections by non-*albicans Candida*, especially azole-resistant *Candida* spp. [25,26]. The shift to non-*albicans Candida* species is also significant because of the newest class of antifungal agents, so the caspofungin, are not currently recommended as primary therapy against *C. parapsilosis*, the third most common species.

Few studies have conducted long-term research on invasive *Candida* in China. The antifungal resistance rate in our study was higher than that reported by Guiyang in 2009 [27] and Hong Kong in 2011 [28]. However, none of the isolates in this study were resistant to caspofungin and amphotericin B. More than 90% of the *C. albicans* isolates were susceptible to all antifungal drugs between 2006 and 2011. We found that fluconazole resistance in *C. albicans* increased from 2.2% to 6.6% and in *C.tropicalis,* increased from 5.1% to 10.6%. There was also,a trend toward increased resistance to azoles in an Italian study [29]. We also found cross-resistance in our study, in agreement with previous reports from other countries [9,30]. A surveillance from 41 institutions participated study had shown that resistance to fluconazole was highly predictive of resistance to voriconazole [22]. In our study, all of the *C. tropicalis* isolates resistant to FLC (MIC ≥ 8 μg/ml) were also resistant to VRC (MIC ≥ 1 μg/ml). The same result in *C. glabrata* isolates which had FLC MIC ≥ 64 μg/ml and VRC ≥ 8 μg/ml was also observed. Therefore, we inferred that resistance to fluconazole highly tend to predictive of voriconazole resistance for *C. glabrata* and *C. tropicalis*.

Fifty-four CAI patterns were recognized from the 136 *C. albicans* isolates. CAI SSCP genotyping showed that the *C. albicans* strains were polymorphic. *C. albicans* isolates in different samples from the same patient had the same genotype. Compared to Li’s study [13], we found more patterns. These results implied that the genotypic distribution of *C. albicans* strains may be various in different samples. Isolates in different samples from the same patient showed exactly the same CAI SSCP pattern (e.g. isolates from patients CY61, CY65, CY66, CY69, CY141, CY119, CY123, CY68 and CY154 in Table 2), indicating that the infection was mainly endogenous [14,31].

**Table 2:** Different specimens, isolation times, and genotypes of clinical samples

**No. patient**	**Isolation time**	**Specimen**	CAI* genotype
CY61A	2010-08-20	blood	25-35
CY61B	2010-08-20	ascitic fluid	25-35
CY65A	2010-02-25	blood	11-20
CY65B	2010-02-25	catheter fluid	11-20
CY66A	2010-03-30	blood	21-21
CY66B	2010-03-29	catheter fluid	21-21
CY69A	2010-08-20	blood	16-21
CY69B	2010-08-23	catheter fluid	16-21
CY141A	2011-01-02	blood	32-46
CY141B	2011-01-07	catheter fluid	32-46
CY119A	2010-01-10	blood	16-18
CY119B	2010-01-07	ascitic fluid	16-18
CY123A	2010-04-14	ascitic fluid	17-28
CY123B	2010-04-14	catheter fluid	17-28
CY68A	2010-03-23	blood	25-33
CY68B	2010-03-20	catheter fluid	25-33
CY68C	2010-03-22	ascitic fluid	25-33
CY154A	2011-09-26	blood	16-21
CY154B	2011-09-26	catheter fluid	16-21
CY154C	2011-10-01	bile	16-21
CY154D	2011-10-04	ascitic fluid	16-21

*CAI CA is the abbreviation of *Candida albicans*, followed by the roman numeral I.

## Conclusions

In summary, this study showed the epidemiology and antifungal resistance of invasive *Candida* spp*.* isolated from Beijing Chaoyang hospital between 2006 and 2011. *C. albicans* was the most frequently isolated species, compared to the non-*albicans Candida* spp. Although the overall rates of resistance to antifungal agents remain low, species-specific raise some concerns, especially *C. tropicalis* and *C. glabrata* were the most frequent detected among organisms resistant to fluconazole. Currently, Caspofungin and amphotericin B are highly effective *in vitro* against all *Candida* species. Most of the invasive candidiasis cases occurred in the ICU, so careful attention should be paid to ICU patients to prevent them from contracting this disease. Genotyping of CAI SSCP showed that the *C. albicans* strains were polymorphic from blood and sterile samples and isolates from different samples of the same patient had the same genotype.

## Abbreviations

CAI SSCP: Single-strand conformation polymorphism; ICU: Intensive care unit; MIC: Minimal inhibitory concentration; R: Resistant.

## Competing interests

The authors declare that they have no competing interests.

## Pre-publication history

The pre-publication history for this paper can be accessed here:

http://www.biomedcentral.com/1471-2334/13/353/prepub
